# Laryngeal Myxoma: A rare localization

**DOI:** 10.22038/IJORL.2021.54133.2847

**Published:** 2022-01

**Authors:** Malek Mnejja, Imen Achour, Wadii Thabet, Rania Kharrat, Rim Kallel, Tahia Boudawara, Bouthaina Hammami, Ilhem Charfeddine

**Affiliations:** 1 *Department of Otorhinolaryngology, Habib Bourguiba Hospital, University of Sfax, Sfax, Tunisia. *; 2 *Department of Pathology, Habib Bourguiba Hospital, University of Sfax, Sfax, Tunisia.*

**Keywords:** Larynx, Myxoma, Polyp, Vocal cord

## Abstract

**Introduction:**

Myxomas are benign mesenchymal neoplasms which arise mainly in the heart. The laryngeal localization is very rare. We aim to describe the clinical, histological and therapeutic features of this condition.

**Case Report:**

We report two cases of laryngeal myxomas occurred in male and female patients, presenting with a history of prolonged hoarseness. Laryngoscopy revealed a polypoid mass on the true vocal folds. The lesions were excised with cold instruments. One patient presented a recurrence 4 years after the first surgery.

**Conclusions:**

Laryngeal myxoma should be considered in case of a benign looking vocal fold lesion, especially a vocal cord polyp. Histologic exam is the only tool to confirm the diagnosis. It is treated by surgical resection. In the literature, recurrence is rare in laryngeal site, but patients need to be kept on close follow-up.

## Introduction

Myxoma is a rare myxoid tumor. It interests mostly the heart and occurs rarely in the head and neck region (1). The laryngeal localization is exceptional. Myxoma is a benign tumor with a propensity for local infiltration and recurrence (2). In this article, we report two new cases of laryngeal myxomas. The purpose of this paper is to describe the clinical, histological and therapeutic features of this condition.

## Case Report

***Case 1***

**Fig 1:**
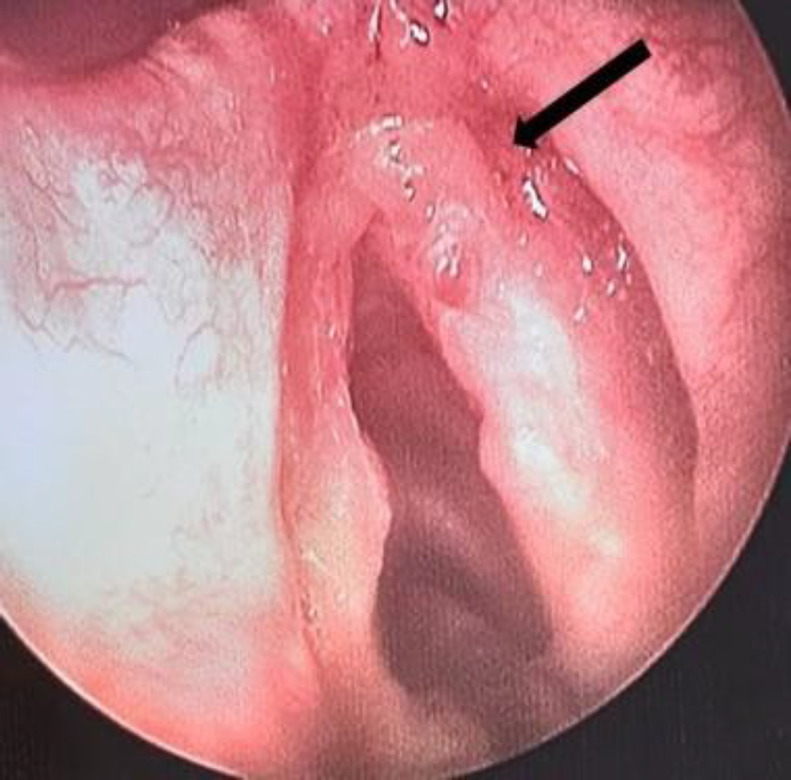
Direct laryngoscopy shows polypoidal lesion on the right vocal cord (black arrow)

**Fig 2:**
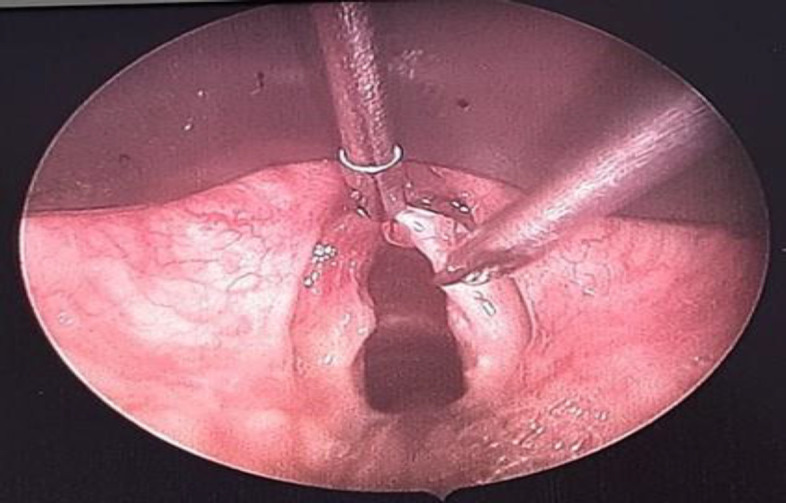
Excision of the lesion with cold instruments

A 59-year-old man with no past medical history was referred to our outpatient clinic with a 2-year history of intermittent hoarseness. He was a smoker with no history of voice abuse. On laryngoscopic examination, there was a polypoidal lesion located in the anterior half of the right true vocal fold with preserved mobility (Figure 1). The lesion was excised with cold instruments: cordectomy (type II) (Figure 2). Histological examination showed a subepithelial paucicellular lesion, which formed by regular spindle to stellate fibroblast cells. These latter were embedded within an abundant myxoid matrix. The tumor was richly vascularized. No histological signs of malignancy were found. These features were consistent with a diagnosis of laryngeal myxoma. After 4 years of follow-up, he had a recurrence located in the same site as the first lesion. He underwent type III cordectomy by cold instruments.

***Case 2***

**Fig 3:**
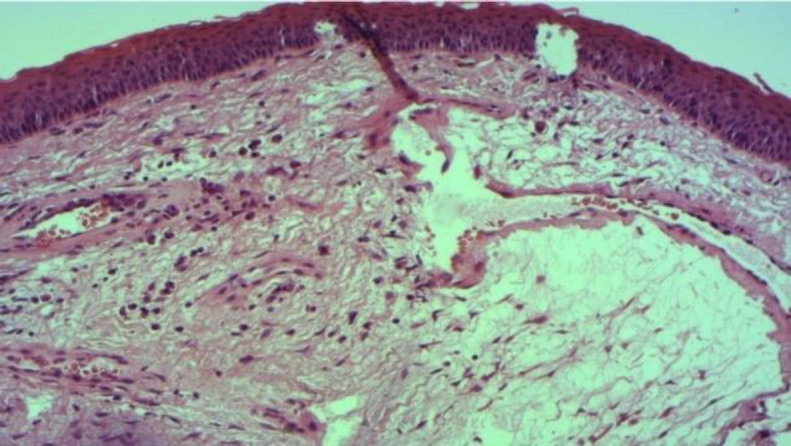
A subepithelial paucicellular lesion, which formed by spindle to stellate cells within an abundant and hypovascular myxoid stroma (Hematoxylin and Eosin x 50)

A 48-year-old woman with no past medical history presented with hoarseness of 20-year duration. She was a smoker with history of voice abuse. Laryngoscopic examination showed a polypoidal mass located in the anterior half of the two true vocal folds. Rest of the vocal cords was edematous. Vocal cord mobility was normal. She had also a thyroid nodule (size: 2 cm / EUTIRADS (European Thyroid Imaging Reporting and Data System) 5). She underwent right lobo-isthmectomy and direct laryngoscopy with excision of the laryngeal lesion by cold instruments. Histological findings were the same as the first case but the lesion was poorly vascularized in this case: a subepithelial paucicellular lesion, which formed by regular spindle to stellate fibroblast cells within an abundant and hypovascular myxoid stroma. No histological signs of malignancy were found. The lesion was lined by a normal stratified squamous epithelium (Figure 3). The diagnosis of laryngeal myxoma was established. Anatomopathological exam of the thyroid revealed a benign multinodular goiter. After 4 years of follow-up, she exhibited no evidence of recurrence.

## Discussion

Myxoma is a myxoid benign neoplasm of mesenchymal origin (1-3). It may arise in any anatomic site but it mainly occurs in the heart (1-3). Myxomas rarely affect the head and neck district, where they generally have an odontogenic origin. Thus, they mostly involve the mandible and the maxilla (1,2,4-6). The laryngeal site is extremely rare (1,2,6). Laryngeal myxoma mostly affects men in the 5th–6th decade with a history of smoking (1,2,5). Our two patients were smokers. The etiopathogenesis of laryngeal myxoma remains unknown. Immature fibroblasts could be responsible for the occurrence in the larynx: modified fibroblastic cells not able to polymerize collagen, so, they produce a large amount of glycosaminoglycans, as an alternative. This amount of glycosaminoglycans gives myxomas a gelatinous appearance on macroscopic examination (2,5,7). Due to the preponderance in the smoking patients, a role for smoking in the pathogenesis of laryngeal myxomas could be proposed (1). Myxomas can be associated with Reinke’s edema (2). However, possibility of Reinke’s edema being a precursor lesion or sharing the same etiopathogenesis with myxoma needs to be studied in detail (2). 

Clinical presentation is similar to common benign fold disorders (laryngeal polyp, cyst, nodule…) (2). Hoarseness is the most common presenting symptom (2,5,8). A large myxoma may cause dyspnea (1). The clinical appearance of laryngeal myxomas is very similar to a laryngeal polyp (1-3,6). The most common location is true vocal folds (1). In our two cases, myxoma was located in the true vocal cords. Macroscopically they appear as grey-white, usually well circumscribed masses with a jelly cut surface (1,5,9). Myxoma is characterized histologically by bland, spindle to stellate cells within an abundant and hypovascular myxoid stroma (2,5,7) It is mostly hypocellular (1). The cellular variant shows hypercellularity, more numerous collagen fibers, and increased vascularity (7). 

Myxoma lacks a fibrous capsule, so it tends to infiltrate the surrounding tissue (2,3,5). There is no general consensus regarding myxoma-specific immunohistochemistry markers (2). True vocal fold myxomas are often found in the superficial lamina propria (9). 

The main differential diagnosis includes some frequently encountered benign lesions, such as vocal cord polyps, but also several malignancies, such as myxoid liposarcoma, myxoid leiomyosarcoma, myxoid chondrosarcoma, rhabdomyosarcoma, myxoid neurofibroma, low-grade myxofibrosarcoma and low-grade fibromyxoid sarcoma (1,7,8). The absence of stromal vasculature, hemorrhage, hemosiderin-laden macrophages, and hyalinization of basement membrane helped to differentiate myxoma from a vocal fold polyp (2,8). 

Moreover, immunohistochemistry is very helpful in assisting diagnosis: Myxoma cells are negative for desmin, actin and S100 protein (1,8). Excision by cold instruments was the preferred approach (2). In our two cases, myxoma was excised with cold instruments.

The progression of myxomas is very slow (3,5,6). Despite their benignity, myxomas are characterized by local infiltration and recurrence if not excised with margins (2,4). However, unlike myxomas originating outside the larynx, recurrence is not widely described in cases of laryngeal myxoma (2). To the best of our knowledge, there is one reported recurrence of laryngeal myxoma (4). One of our two patients presented a recurrence 4 years after the first surgery. So, Panda et al think that microlaryngeal surgery performed from the point of view of a vocal fold polyp or cyst will generally suffice (2). Mesolella et al and Tang et al think that treatment gold standard consists in a wide and complete resection with free surgical margins to prevent its reappearance (1,4), but as much as possible conservative to obtain an optimal postoperative vocal function.

No malignant transformation of laryngeal myxoma has been reported.

## Conclusion

Although laryngeal myxoma is an infrequent condition, it should be considered in case of a benign looking vocal fold lesion, especially a vocal cord polyp, as in our two cases. Hoarseness is the most common presenting symptom. Histologic examination is an important tool to obtain final diagnosis and differentiation from other possibilities. Laryngeal myxoma is treated by surgical excision. Resection by cold instruments is the preferred approach. In our two cases, myxoma was excised with cold instruments. After surgery, the rate of recurrence is low comparing with myxomas originating outside the larynx. However, patients need to be kept on close follow-up to rule out recurrence. One of our two patients presented a recurrence 4 years after the first surgery.
